# Cardiac magnetic resonance using late gadolinium enhancement and atrial T1 mapping predicts poor outcome in patients with atrial fibrillation after catheter ablation therapy

**DOI:** 10.1038/s41598-018-31916-2

**Published:** 2018-09-11

**Authors:** Julian A. Luetkens, Anne C. Wolpers, Thomas Beiert, Daniel Kuetting, Darius Dabir, Rami Homsi, Hendrik Meendermann, Natalie Abou Dayé, Vincent Knappe, Morten Karsdal, Signe H. Nielsen, Federica Genovese, Florian Stöckigt, Markus Linhart, Daniel Thomas, Georg Nickenig, Hans H. Schild, Jan W. Schrickel, René P. Andrié

**Affiliations:** 1 Department of Radiology, University Hospital Bonn, Rheinische Friedrich-Wilhelms University, Bonn, Germany; 2 Department of Internal Medicine II, University Hospital Bonn, Rheinische Friedrich-Wilhelms University, Bonn, Germany; 3 Fibrosis Biology and Biomarkers, Nordic Bioscience, Herlev, Denmark

## Abstract

To determine the pre-procedural value of different fibrotic biomarkers and comprehensive cardiac magnetic resonance (CMR) for the prediction of poor response to ablation therapy in patients with atrial fibrillation (AF). Left atrial (LA) late gadolinium enhancement (LGE) and native LA T1 relaxation times were assessed using CMR. Plasma levels of relaxin, myeloperoxidase and serum levels of matrix metalloproteinase (MMP)-mediated cardiac specific titin fragmentation and MMP-mediated type IV collagen degradation were obtained. Poor outcome was defined by the recurrence of AF during 1-year follow-up. 61 patients were included in final analysis. Twenty (32.8%) patients had recurrence of AF. Patients with a recurrence of AF had a higher percentage of LA LGE (26.7 ± 12.5% vs. 17.0 ± 7.7%; P < 0.001), higher LA T1 relaxation times (856.7 ± 112.2 ms vs. 746.8 ± 91.0 ms; P < 0.001) and higher plasma levels of relaxin (0.69 ± 1.34 pg/ml vs. 0.37 ± 0.88 pg/ml; P = 0.035). In the multivariate Cox regression analysis, poor ablation outcome was best predicted by advanced LGE stage (hazard ratio (HR):5.487; P = 0.001) and T1 relaxation times (HR:1.007; P = 0.001). Pre-procedural CMR is a valuable tool for prediction of poor response to catheter ablation therapy in patients with AF. It offers various imaging techniques for outcome prediction and might be valuable for a better patient selection prior to ablation therapy.

## Introduction

Atrial fibrillation (AF) remains the most common cardiac arrhythmia and is characterized by an irregular ventricular interval and the absence of distinct organized atrial activity on electrocardiogram^1^. Although the underlying pathophysiology is still incompletely understood, several studies suggest that profibrotic and inflammatory processes play a pivotal role in the mechanisms of AF^2,3^. Furthermore, the development and recurrence of atrial fibrillation is tightly linked to a fibrotic remodeling of the left atrium^1,4^. As pulmonary vein ectopic activity has been linked to the initiation and maintenance of AF, pulmonary vein isolation (PVI) is used to cut the electric link between pulmonary veins and the left atrium. The procedure helps to restore normal sinus rhythm and has become a widely accepted treatment strategy in patients with paroxysmal and persistent AF^2–4^. However, as success rates of PVI differ widely, there is a special need for conclusive preprocedural parameters for the reliable identification of patients who have a low chance of ablation success and therefore might be considered not eligible for AF ablation.

Recently, cardiac magnetic resonance (CMR) imaging using a late gadolinium enhancement (LGE) sequence has been described to be independently associated with the likelihood of recurrent arrhythmia after PVI^5,6^. In left atrial (LA) LGE CMR, the amount of contrast enhancement in the LA wall is considered to represent the volume of fibrotic tissue of the total LA wall^6,7^. A higher amount of atrial fibrotic tissue can favor atrial arrhythmogenesis^6,7^. Recently, more severe fibrosis detected via LGE CMR was also linked to an increased risk of stroke or transitory ischemic attack^8^. Another, newer CMR technique for noninvasive tissue characterization based on the quantitative calculation of myocardial T1 relaxation times is myocardial T1 mapping. To date, myocardial T1 mapping has been used for tissue characterization of the left ventricular myocardium and seems to be a promising new tool for the assessment of myocardial fibrosis and edema^9,10^. Several studies revealed high correlations of myocardial T1 relaxation times with histological collagen volume fraction of the left ventricle^9,11^.

Beside imaging parameters, inflammatory pathways have been described as a crucial initiator of LA fibrosis. Several inflammatory serum biomarkers, like profibrotic myeloperoxidase, are associated with a higher risk of AF recurrence after PVI^12,13^. In experimental models, matrix metalloproteinase (MMP)-mediated cardiac specific titin fragmentation (TIM) and MMP-mediated type IV collagen degradation (C4M) are a reflection of cardiac tissue remodeling, whereas relaxin seems to have anti-fibrotic properties in the development of AF^14–17^. Chronic inflammatory processes might therefore be a prequel of altered deposition of extracellular matrix in LA fibrosis, which can conclusively be visualized on CMR.

The aim of this prospective study was, therefore, to investigate the pre-procedural value of different fibrotic biomarkers and comprehensive CMR (including LGE and native T1 mapping) for the prediction of poor response to ablation therapy.

## Materials and Methods

The responsible ethics committee of the university of Bonn approved this study, and all subjects gave informed consent. All experiments were performed in accordance with relevant guidelines and regulations. This prospective study included patients with paroxysmal or persistent AF scheduled for first PVI by cryoballoon ablation (second generation) at our institution between February 2015 and January 2016. Patients with long standing persistent AF were excluded. All patients had transthoracic and transesophageal echocardiography prior to PVI. Blood samples and LA CMR were conducted at least 4 days prior to PVI. A 72 holter monitor test performed at 3, 6, 9 and 12 months after PVI. The study endpoint was the recurrence of AF (documented arrhythmia episodes (>30 sec)) during 1-year follow-up after a 90-day blanking period.

### Cardiac magnetic resonance

CMR scans were performed on a 1.5 Tesla CMR system (Ingenia 1.5 T, Philips Healthcare, Best, The Netherlands). LA native T1 mapping was performed in end-systole in transversal orientation using a high-resolution 3(3)3(3)5 modified Look-Locker inversion recovery (MOLLI) acquisition scheme^18^ (acquisition matrix: 320 × 320 mm; time of repetition (TE): 2.18 ms; time of echo (TE): 1.02 ms; parallel imaging factor (SENSE): 2; voxel size (acquired): 2.00 × 2.00 × 8 mm; voxel size (reconstructed): 1.17 × 1.17 × 8 mm; flip angle: 35°; estimated scan duration/breath-hold: 00:15 min). For atrial (LGE) imaging a high-resolution ECG-triggered and navigator gated 3D inversion recovery was performed in transversal orientation covering the entire left atrium. Sequence parameters were as follows: acquisition matrix: 300 × 240 mm; time of repetition (TE): 3.6 ms; time of echo (TE): 1.8 ms; voxel size (acquired): 1.3 × 1.3 × 5.0 mm; voxel size (reconstructed): 0.74 × 0.74 × 2.5 mm; flip angle: 15°; estimated scan duration: 01:54 min. LGE images were acquired 15 minutes after injection of a bolus of 0.2 mmol/kg of body weight of gadobutrol (Gadovist, Bayer Healthcare, Leverkusen, Germany). Optimal inversion time was determined by using the Look-Locker technique^19^.

T1 maps were reconstructed offline using a dedicated plugin for the OsiriX DICOM viewer software (Pixmeo, Geneva, Switzerland). For motion correction, the LA posterior wall was manually defined via polygon regions of interests and carefully realigned throughout the MOLLI relaxometry data. Then, an exponential fitting with a maximum likelihood estimator was used to calculate the T1 maps. The following fit model was used for the magnitude data:

$$|{\rm{A}}+{\rm{B}}\times \exp (-{\rm{TI}}/{\rm{T1}}\ast )|.$$

A Rician noise distribution was assumed because of the magnitude operation. A Look–Locker correction was performed to calculate T1 based on the fit parameters T1*, A, and B. LA T1 relaxation times were extracted from the relaxation maps via regions of interest analysis.

For LGE analysis, the margins of the left atrium were carefully manually traced throughout all available transversal slices via multiple regions of interest analysis (width: 1.477 mm) using Image J (National Institute of Mental Health, Bethesda, Maryland, USA). Positive LA enhancement on LGE images was assessed as previously described using an individual threshold for fibrosis identification^5^. The total volume of positive LGE (fibrotic tissue) is calculated as a percentage of LA wall volume. Depending on the percentage of total wall enhancement patients were categorized into four fibrosis stages: Utah I (<10% fibrosis), Utah II (10–20% fibrosis), Utah III (20–30% fibrosis), and Utah IV (>30% fibrosis).

### Laboratory parameters

Laboratory parameters were determined at baseline. Most parameters were evaluated in the clinical laboratory as per routine clinical practice. Relaxin-2 and myeloperoxidase were determined using commercial available solid-phase Quantikine ELISA (R&D Systems, Minneapolis, Minnesota, USA). The concentration of MMP-mediated titin and collagen type IV degradation fragments was determined by competitive ELISAs (Nordic Bioscience, Herlev, Denmark). The measurements were performed as previously described^14,17^.

### Cryoballoon pulmonary vein isolation

Venous access for PVI was obtained through the right femoral vein. Having positioned a decapolar catheter over a 7 F sheath in the coronary sinus, next a single transseptal puncture (Brockenbrough technique) was performed under fluoroscopic guidance. For selective angiography of the individual pulmonary veins a 15 F sheath (Flexcath^®^, Medtronic, Inc., Minneapolis, Minnesota, USA) was used. After angiography, a 28 mm second generation cryoballoon (Arctic Front Advance^®^; Medtronic, Inc., Minneapolis, Minnesota, USA) was positioned in front of the PV ostium with best possible occlusion, controlled by fluoroscopy. Cryoenergy was applied twice for a period of four minutes each. Prior to isolation of the right superior PV (RSPV), the decapolar catheter was removed from the coronary sinus and positioned in the superior vena cava for continuous stimulation of the phrenic nerve during application of cryoenergy. Recognition of diminishing movements of the diaphragm during fluoroscopy led to instantaneous termination of cryoapplication in the RSPV.

Ablation success was verified using a spiral mapping catheter (AchieveTM, Medtronic, Inc., Minneapolis, Minnesota, USA) advanced in the individual targeted PV. If remaining ostial PV potentials were recorded, the respective vein was treated again with the cryoballoon. During the procedure patients were continuously anticoagulated with heparin (targeted activated clotting time: 350 s). After the procedure, pericardial effusion was excluded via transthoracic echocardiography. The following day a 12-lead ECG was recorded before discharge from hospital. Anticoagulation was managed according to guideline recommendations^20^.

### Statistical analysis

Statistical analysis was performed using SPSS Statistics 22.0 (IBM, Armonk, NY, USA) and MedCalc 11.0 (MedCalc Software bvba, Ostend, Belgium). Patient characteristics are presented as mean ± standard deviation or as absolute frequency. Continuous variables were checked for normal distribution. The independent 2-sample Student *t* test (for normally distributed variables) or the Mann–Whitney U test (for not normally distributed variables) was used for comparison of continuous variables between 2 different groups. Dichotomous variables were compared using the χ2 test (with a cell count >5) or Fisher exact test (with a cell count ≤5). Correlation analysis was performed using Spearman’s rank correlation coefficient. Significant independent predictors of AF recurrence were analyzed by univariate and subsequent multivariate Cox regression analysis. Results are displayed as adjusted hazard ratio (HR) with 95% confidence interval (CI). The level of statistical significance was set to P < 0.05.

## Results

### General characteristics

**Figure 1:**
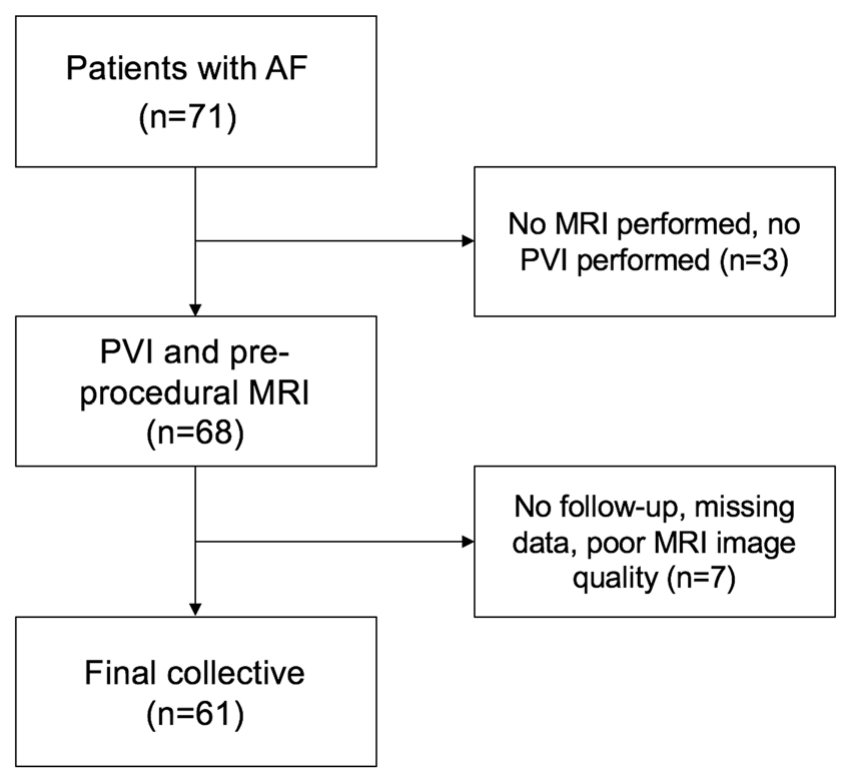
Study flow chart showing number of patients included in this study. The final collective consisted of 61 patients.

A total of 71 patients were enrolled in this study. 10 (14.1%) patients were excluded due to missing data or poor CMR quality (see Fig. 1). 40 (65.6%) of all patients had paroxysmal AF, whereas 21 (34.4%) of all patients had persistent AF. Mean AF duration prior to ablation therapy was 52.0 ± 66.8 months. After single PVI procedure, AF recurred in 20 (32.8%) patients during the 1-year follow-up. Early recurrence during the 90 days blanking period after the procedure occurred in 14 (23.0%) of all patients. From these 14 patients, 10 (71.4%) patients had a recurrence of AF during 1-year follow-up. For those who experienced recurrent arrhythmia within the one year follow-up, the median time to recurrence was 207 (range: 92–364) days. Mean age of patients with recurrence was 58.5 ± 13.3 (range: 23–80) years. Mean age of patients without recurrence of AF was 61.2 ± 2.2 (range: 28–83) years. Age (P = 0.554), sex (P = 0.453), and body mass index (P = 0.933) did not differ significantly between both groups. Detailed clinical characteristics for patients with and without recurrence of AF are given in Table 1.

**Table 1:** Clinical characteristics in patients with and without recurrence of atrial fibrillation after ablation therapy.

Variable		No recurrence (n = 41)	Recurrence (n = 20)	P-Value
Age (years)		61.2 ± 12.2	58.5 ± 13.3	0.554
Female (n)		14 (34.1%)	7 (35.0%)	0.453
BMI (kg/m²)		28.4 ± 4.3	29.7 ± 5.7	0.933
CHA_2_DS_2_VASc score	0–1	15 (36.6%)	10 (50.0%)	0.317
	>1	26 (63.4%)	10 (50.0%)	0.253
HAS-BLED score	0–2	34 (82.9%)	19 (95.0%)	0.253
	>2	7 (17.1%)	1 (5.0%)	0.190
Paroxysmal AF type (n)		29 (70.7%)	11 (55.0%)	0.225
Left atrial volume (ml)		51.6 ± 19.6	62.5 ± 28.8	0.051
Mitral valve insufficiency	Stage 0-II	27 (65.9%)	15 (75.0%)	0.226
	Stage III	14 (34.1%)	5 (25.0%)	0.226
Obstructive sleep apnea		5 (12.2%)	4 (20.0%)	0.461
Coronary artery disease		9 (22.0%)	3 (15.0%)	0.734
*Cardiovascular risk factors*				
Diabetes mellitus (n)		3 (7.3%)	0 (0.0%)	0.544
Hyperlipidemia (n)		19 (46.3%)	5 (25.0%)	0.109
Nicotine abuse (n)		15 (36.6%)	6 (30.0%)	0.611
Family disposition (n)		15 (36.6%)	8 (40.0%)	0.769
*Cardiac medications*				
Beta-Blocker		32 (78.0%)	16 (80.0%)	0.999
Class I agents		10 (24.4%)	6 (30.0%)	0.758
Class III agents		4 (9.7%)	3 (15.0%)	0.384
ACE inhibitor		21 (51.2%)	10 (50.0%)	0.929
Aldosterone antagonist		0 (0.0%)	1 (5.0%)	0.328
Calcium antagonist		6 (14.6%)	1 (5.0%)	0.409
Digitalis		2 (4.9%)	2 (10.0%)	0.591
Diuretics		11 (26.8%)	7 (35.0%)	0.511

Data are mean ± standard deviation or absolute frequency with percentages in parentheses. BMI = body mass index; AF = atrial fibrillation; ACE = angiotensin converting enzyme.

### Cardiac magnetic resonance

**Figure 2:**
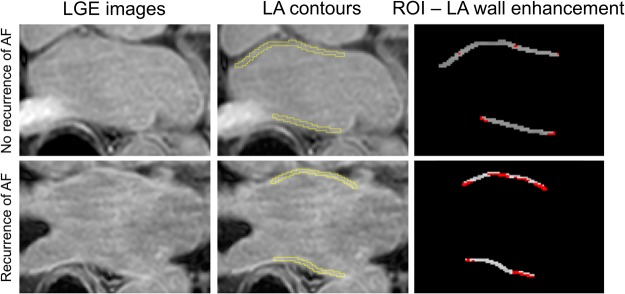
Late gadolinium enhancement (LGE) images of a patient with recurrence and without recurrence of atrial fibrillation (AF). Contours were manually drawn at the epicardial and endocardial borders of the left atrial (LA) wall. For analysis, all LA wall region of interests (ROIs) were cropped and an individual threshold intensity was applied per slice, which was likely to correspond to the enhanced/fibrotic voxels (red colored voxels) of the LA wall. Fibrotic voxels were determined as followed: In a first step, the lower region of the pixel intensity histogram of the LA ROIs (between 2% and 40% of the maximum intensity) were defined as “normal”. In a second step, the fibrotic threshold was then calculated as two to four standard deviations above the mean of “normal” and checked for appropriateness with the original LGE images. The most used cutoff was three standard deviations. The patient with no recurrence of AF had a left atrial wall enhancement of 10.2%. The patient with recurrence had an enhancement of 43.6%.

**Figure 3:**
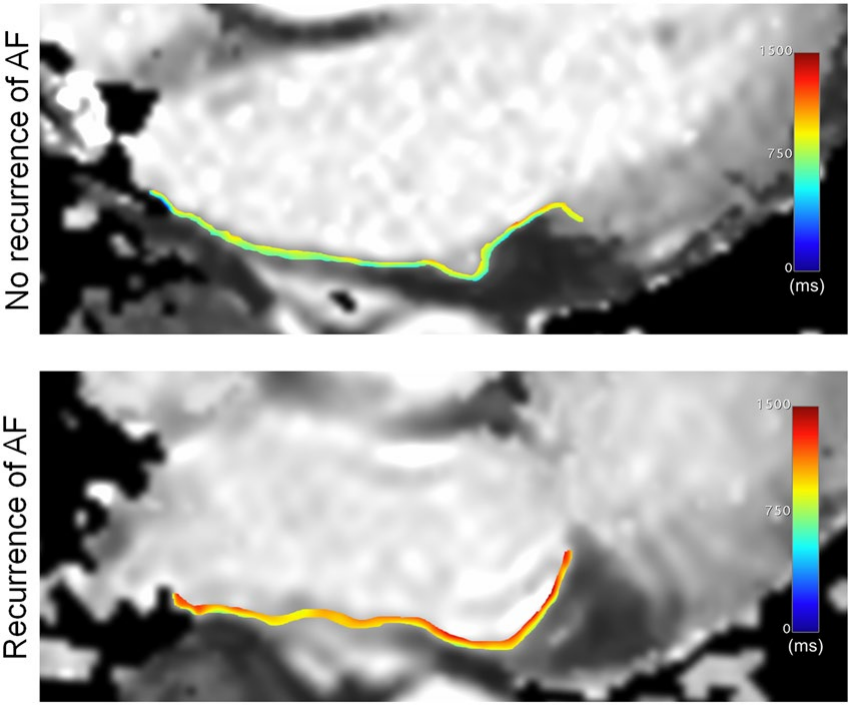
Example of T1 relaxation time measurements of the left atrial posterior wall. T1 relaxometry data was measured via region of interest (ROI) analysis. For better visualization, T1 relaxation times within the ROIs are color-coded. The patient with no recurrence of atrial fibrillation had a left atrial T1 relaxation time of 662 ms. The patient with recurrence had a left atrial T1 relaxation time of 915 ms. AF = atrial fibrillation.

**Figure 4:**
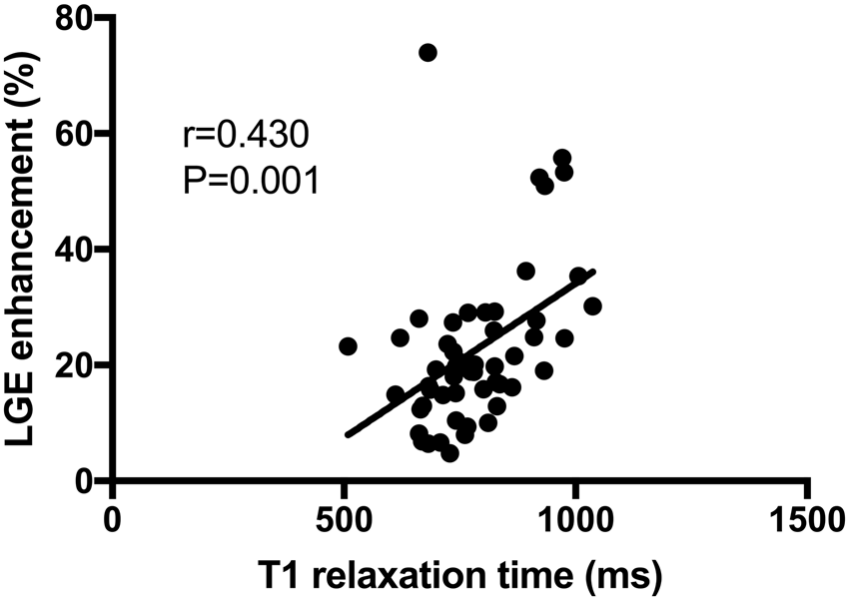
Scatter plot showing correlation between percentage of atrial wall enhancement on late gadolinium enhancement (LGE) images and left atrial T1 relaxations times.

85.2% (52/61) of all CMR scan were performed in sinus rhythm. The percentage of LA wall enhancement on LGE CMR was significantly greater in the patient group with AF recurrence compared to the group without recurrence (26.7 ± 12.5% vs. 17.0 ± 7.7%; P < 0.001) (see Table 2 and Fig. 2). Native LA T1 relaxation times were also significantly increased in patients with recurrence of AF compared to patients without recurrence (856.7 ± 112.2 ms vs. 746.8 ± 91.0 ms; P < 0.001) (see Fig. 3). There was a significant positive correlation between LA LGE wall enhancement and LA T1 relaxation times (r = 0.430; P = 0.001) (see Fig. 4). All patients with AF were classified into four fibrosis stages based on the percentage of LA wall enhancement on LGE CMR as follows: 11 (18.0%) Utah stage I, 25 (41.0%) Utah stage II, 16 (26.2%) Utah stage III, and 9 (14.8%) Utah Stage IV.

**Table 2:** Laboratory parameters and CMR parameters in patients with and without recurrence of atrial fibrillation after ablation therapy.

Variable	No recurrence (n = 41)	Recurrence (n = 20)	P-Value
*CMR parameters*			
Extent of LA wall enhancement (%)	17.0 ± 7.7	26.7 ± 12.5	<0.001
Utah stage I-II	31 (76%)	5 (25%)	<0.001
Utah stage III-IV	10 (24%)	15 (75%)	<0.001
T1 relaxation time (ms)	746.8 ± 91.0	856.7 ± 112.2	<0.001
*Laboratory parameters*			
NT-proBNP (pg/ml)	1119.3 ± 2744.0	902.0 ± 1105.5	0.742
Creatinine (mg/dl)	0.99 ± 0.26	0.96 ± 0.17	0.663
Relaxin (pg/ml)	0.37 ± 0.88	0.69 ± 1.34	0.035
Myeloperoxidase (ng/ml)	38.7 ± 54.4	29.2 ± 19.9	0.778
White blood cell count (G/l)	6.8 ± 1.5	7.7 ± 1.4	0.013
Neutrophils (G/l)	4.1 ± 1.5	4.9 ± 1.1	0.005
CRP (mg/l)	4.8 ± 9.4	3.4 ± 3.6	0.926
PCT (µg/l)	0.03 ± 0.03	0.04 ± 0.03	0.206
IL-6 (pg/ml)	2.5 ± 3.5	3.9 ± 12.5	0.294
IL-8 (pg/ml)	6.7 ± 6.0	6.7 ± 4.4	0.486
C3 (g/L)	0.2 ± 0.1	0.3 ± 0.1	0.049
C4 (g/L)	4.8 ± 0.7	5.0 ± 0.5	0.288
MMP-mediated cardiac specific titin (TIM)-(ng/ml)	311.9 ± 104.5	321.0 ± 122.2	0.927
MMP-mediated type IV collagen degradation (C4M2) (ng/ml)	16.5 ± 4.7	14.3 ± 4.6	0.078

Data are mean ± standard deviation or absolute frequency with percentages in parentheses. LA = left atrial; MMP = matrix metalloproteinase; CMR = cardiac magnetic resonance.

### Laboratory parameters

Patients with recurrence of AF had higher serum concentration of relaxin compared to patients without recurrence of AF (0.69 ± 1.34 pg/ml vs. 0.37 ± 0.88 pg/ml; P = 0.035). Patients with recurrence of AF had also slightly higher C3 values (0.3 ± 0.1 g/L vs. 0.2 ± 0.1 g/L; P = 0.049). No significant group differences were found for myeloperoxidase (29.2 ± 19.9 ng/ml vs. 38.7 ± 54.4 ng/ml; P = 0.778) between both groups. Serum levels of MMP-mediated cardiac specific titin and MMP-mediated type IV collagen degradation fragments did not significantly differ between both groups (see Table 2).

Patients with increased LA LGE enhancement (Utah stage III-IV) had higher levels of myeloperoxidase (30.6 ± 13.4 ng/ml vs. 21.6 ± 10.7 ng/ml; P = 0.046) and a tendency of higher levels of relaxin (0.71 ± 1.38 pg/ml vs. 0.31 ± 0.69 pg/ml; P = 0.075) compared to patients with Utah stage I-II. The white blood count (7.7 ± 1.5 G/l vs. 6.7 ± 1.5 G/l; P = 0.016) as well as the neutrophils (5.0 ± 1.2 G/l vs. 3.9 ± 1.4 G/l; P = 0.002) were significantly associated to higher levels of LA wall enhancement.

### Impact of different parameters on freedom of atrial fibrillation

**Figure 5:**
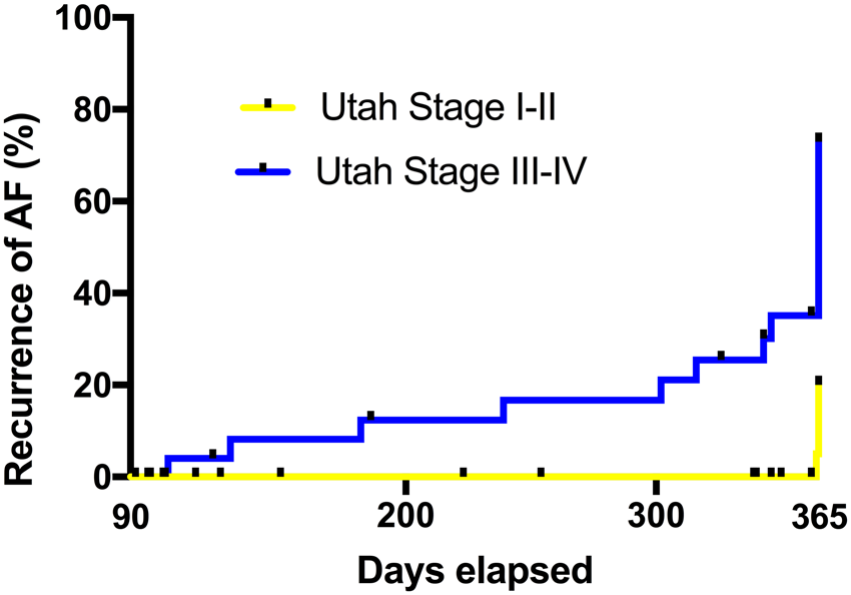
Cumulative incidence of arrhythmia recurrence after blanking period for low and high stages of fibrosis. AF = atrial fibrillation.

In the univariate Cox regression analysis T1 relaxation time (HR 1.008 (95% CI: 1.003–1.012); P < 0.001) and higher fibrosis stages on LGE CMR (HR 5.811 (95% CI: 2.105–16.038); P = 0.001) were associated with arrhytmia recurrence. Also, white blood cell count (HR 1.370 (95% CI: 1.038–1.809); P = 0.026) was associated with arrhythmia recurrence. In the multivariate model, only T1 relaxation time (HR 1.007 (95% CI: 1.003–1.011); P = 0.001) and higher fibrosis stages on LGE CMR (HR 5.487 (95% CI: 1.920–15.680); P = 0.001) (see Fig. 5) were independent predictors of arrhythmia recurrence. The results of the univariate and multivariate Cox regressions are summarized in Table 3.

**Table 3:** Predictors of freedom from recurrent atrial arrhythmia.

Variable	Univariate analysis		Multivariate analysis	
	Hazard ratio	P-Value	Hazard ratio	P-Value
Age	0.989 (0.953–1.020)	0.427	—	—
AF type (paroxysmal vs. persistent)	0.627 (0.259–1.514)	0.305	—	—
Left atrial volume	1.018 (0.999–1.018)	0.055	—	—
T1 relaxation time	1.008 (1.003–1.012)	<0.001	1.007 (1.003–1.011)	0.001
Utah stage I-II vs. Utah stage III-IV	5.811 (2.105–16.038)	0.001	5.487 (1.920–15.680)	0.001
Relaxin	1.282 (0.876–1.877)	0.201	—	—
White blood cell count	1.370 (1.038–1.809)	0.026	—	—
Neutrophils	1.029 (0.977–1.083)	0.278	—	—
C3	0.985 (0.977–1.083)	0.992	—	—

Predictors of freedom from recurrent arrhythmia after ablation therapy were determined by using Cox regression. Significant univariate predictors were entered into the multivariate model. Hazard ratios are given with 95% confidence interval. AF = atrial fibrillation.

## Discussion

In this prospective study, we evaluated the pre-procedural value of comprehensive CMR and different serum measurements of fibrotic biomarkers for the prediction of poor response in patient with AF undergoing ablation therapy. The main findings of our study are that (1) both CMR markers of atrial fibrosis (percentage of positive LGE and T1 relaxation times) were significantly elevated in patients with recurrence of AF, (2) some inflammatory and fibrotic biomarkers including anti-fibrotic relaxin also allowed a group discrimination between patients with and without recurrence of AF, and (3) on multivariate analysis T1 relaxation times and higher fibrosis stages were the only independent predictors of poor outcome following ablation therapy.

### Cardiac magnetic resonance

The presence of LA fibrosis has been postulated as a major cause of conduction abnormalities underlying the initiation and maintenance of AF^4^. The development of cardiac and atrial fibrosis is characterized by an increase in collagen and other extracellular matrix components^21^. On LGE CMR, an extracellular contrast agent accumulates in areas with an increased extracellular compartment without entering the myocardial cells and thus focal and patchy areas of atrial and myocardial fibrosis can be visualized^1^. Recently, a prospective multicenter trial (the DECAAF study) reported that atrial tissue fibrosis estimated by LGE CMR was independently associated with the recurrence of AF following PVI^6^. Especially patients with higher fibrosis stages on LGE images (Utah stage III (20–30% fibrosis) and Utah stadium IV (>30% fibrosis)) had an increased risk of recurrence. Another study in 426 patients, partly confirming the results of the DECAAF study, showed that recurrent arrhythmia occurred mainly in patients with higher atrial enhancement on LGE images (recurrence of AF in 71.4% of patients with Utah stage IV)^7^. In our study, we could achieve similar LGE CMR results: higher fibrosis stages (Utah stage III and IV) were independently associated with the risk of PVI failure (HR: 5.487). Our study results therefore support those previous studies showing that pre-existing fibrosis and scarring on LGE CMR are independent predictors of ablation procedure failure and arrhythmia recurrence. This may be because patients with advanced structural fibrotic remodeling, as indicated by LGE CMR, are more likely to have extensive fibrillatory circuits that are difficult to interrupt by PVI, compared to patients with a lower degree of fibrosis/AF substrate. Thus, interruption of the fibrillatory pathways in the early stages of fibrosis seems to be crucial for the success of PVI.

Another CMR technique which has been described to reliably assess myocardial fibrosis is myocardial T1 mapping. T1 mapping allows for a direct myocardial signal quantification according to myocardium’s longitudinal relaxation time^10^. Myocardial T1 relaxation times can be assessed both in the native tissue and after the administration of CMR contrast media (yielding post-contrast T1 relaxation times)^10^. With both T1 mapping approaches it is possible to detect diffuse myocardial fibrosis, which has been demonstrated in multiple histological validation studies of the left ventricle^9,11^. Native T1 values are typically increased in diffuse fibrosis, whereas post-contrast T1 values are decreased (due to the increased accumulation of extracellular gadolinium in areas of fibrosis)^9,11^. The possibility of T1 mapping to detect diffuse fibrosis might be an advantage compared to qualitative/ semi-qualitative LGE imaging, in which the assessment of fibrotic changes might be hindered in cases of diffuse disease (i.e. when no normal signal from healthy, non-fibrotic tissue is present). Compared to LGE imaging for LA fibrosis quantification, T1 mapping has some more advantages: the technique does not require a special software for image analysis, the measurements are less time consuming, and no individual thresholds have to be defined to separate healthy from fibrotic myocardium. Previous studies dealing with T1 relaxation times in patients with AF mainly focused on diffuse left ventricular fibrosis^21–23^. In these studies, post-contrast T1 relaxation times were identified to be decreased in patients with AF compared to healthy controls indicating an association between AF and adverse ventricular remodeling^21,22^. McLellan *et al*. reported shorter post-contrast left ventricular T1 relaxation times to be associated with reduced freedom of AF after PVI^22^. However, another study found left ventricular T1 relaxation time not to be associated with the recurrence of AF after ablation^23^.

To date, only few studies have applied the T1 mapping technique to the left atrium. Beinart *et al*. found post-contrast LA T1 relaxation times to be shorter in patients with AF compared to healthy volunteers^24^. Furthermore, lower LA T1 relaxation times were also associated with lower bipolar LA voltage measurements^24^. Ling *et al*. demonstrated that post-contrast LA T1 relaxation times of <230 ms were associated with a higher risk of arrhythmia recurrence^25^. In contrast to the study of Ling *et al*.^25^, we evaluated LA T1 relaxation times in a more complex multivariate model, which also included several more established (e.g. LA LGE and myeloperoxidase) markers of LA fibrosis and fibrogenesis. Despite this more complex regression model, LA T1 relaxation times were independently associated with PVI failure in our study. This suggests that the assessment of LA T1 relaxation values might be a robust new parameter for the evaluation of LA tissue composition.

Furthermore, in contrast to the study of Ling *et al*.^25^, LA T1 mapping was performed native (i.e. without additional contrast) in our study. In patients with recurrence of AF native LA T1 relaxation times were significantly prolonged. The acquisition of native T1 maps is also associated with some major advantages: First, native T1 mapping avoids possible confounding factor of post-contrast T1 mapping like renal function impairment, hematocrit variations and delay time in measurement after contrast administration^26^. Second, native T1 mapping can also be performed in patients with severe kidney disease and contrast medium allergy. This may be of great importance because the currently proposed CMR protocols for evaluation of LA wall fibrosis (e.g. LGE imaging and post-contrast T1 mapping) are restricted to patients without contraindications for the use of gadolinium-based contrast agents. Another novel finding of our study is that we showed a significant correlation between LA wall enhancement on LGE imaging and LA T1 relaxation times suggesting that both parameters are capable of LA wall fibrosis detection and quantification. In contrast to time consuming LGE LA imaging^6^, a native T1 map can be acquired during a single breath-hold^18^, which might be another advantage, as it would significantly reduce examination time.

### Laboratory parameters

Clinical investigations have reported multiple associations between the vulnerability to AF and increased biomarkers of inflammation^27^. Several inflammatory pathways regulate the homogeneity of the atrial extracellular compartment and are tightly linked to the development of atrial fibrosis^27^. In our study, we found an elevated white blood cell count, a well-established marker of systemic inflammation, in patients with recurrence of AF. These results are in line with multiple previous studies, which also described elevations in white blood cell count and an increased inflammatory cell infiltration in the atrial myocardium in the presence of AF^28,29^. Letsas KP *et al*. also reported the white blood cell count to be significantly associated with AF recurrence after ablation therapy^28^. In our study, white blood cell count was associated with the recurrence of AF only in the univariate regression analysis, but did not reach the level of statistical significance in the multivariate model, possibly due to the strong effect of imaging parameters.

Myeloperoxidase has been described as a progenitor of AF induction that is increased in humans with AF^12^. In animal models, administration of myeloperoxidase is directly linked to atrial fibrotic tissue remodeling^12^. In patients with paroxysmal AF, higher levels of myeloperoxidase are an independent predictor of AF recurrence (HR: 2.12)^13^. In our mixed patient population with paroxysmal and persistent AF, however, we could not reproduce these results. We also tested plasma levels of MMP-mediated protein degradation fragments for the prediction of poor response after PVI. MMPs are a family of functionally related enzymes that cleave matrix components and can promote fibrotic myocardial matrix remodeling^30^. In our cohort, MMP-mediated titin and type IV collagen degradation were not associated with an unfavorable outcome after PVI. On the other hand, we observed higher relaxin levels in patients with AF recurrence. In animal models, relaxin has been described to reduce the susceptibility to AF after myocardial infarction and furthermore can reverse fibrosis in aged rats^15,16^. Interestingly, our data suggests that relaxin seems to be upregulated in patients with higher fibrosis stages. Although relaxin did not allow a prediction of AF recurrence after ablation therapy, the anti-inflammatory and anti-fibrotic properties of relaxin warrants further investigations.

### Limitations

In this study, a systematic endomyocardial biopsy or LA voltage mapping as a reference standard of fibrosis assessment was not performed. Also no previous study correlated LA T1 mapping with histologic fibrosis analysis. However, previous studies reported associations of LGE CMR with histological fibrosis analysis^7^ and associations of LA T1 mapping with left atrial voltage^25^, indicating that the presented CMR techniques are capable to measure LA myocardial fibrosis. MOLLI T1 mapping have been demonstrated to be sensitive to heart rate and rhythm^31^, which could have influenced some measurements, especially in patients without sinus rhythm. Because of the relatively small LA wall thickness and the limited spatial resolution of current CMR techniques, partial volume effects may have led to a non-physiological spread of CMR values when the blood pool or pericardial fat signal intensities might have been included in some cases. Higher resolution CMR imaging will be acquired in future to facilitate a more detailed and comprehensive atrial tissue characterization. Because of the small sample size and the single-center design of the study, further prospective studies are necessary to substantiate the results of this study.

## Conclusion

In this study, we could show that CMR markers of atrial fibrosis (percentage of positive LGE and T1 relaxation times) were significantly elevated in patients with recurrence of AF. Both parameters were independent predictors of poor outcome following ablation therapy. Pre-procedural CMR seems to be a valuable tool for prediction of poor response to catheter ablation therapy in patients with AF. It offers various imaging techniques for outcome prediction and might be valuable for a better patient selection prior to ablation therapy.
